# Antihypertensive drug use and prostate cancer-specific mortality in Finnish men

**DOI:** 10.1371/journal.pone.0234269

**Published:** 2020-06-29

**Authors:** Aino Siltari, Teemu J. Murtola, Kirsi Talala, Kimmo Taari, Teuvo L. J. Tammela, Anssi Auvinen

**Affiliations:** 1 Faculty of Medicine and Health Technology, Tampere University, Tampere, Finland; 2 Department of Pharmacology, Faculty of Medicine, University of Helsinki, Helsinki, Finland; 3 Department of Urology, TAYS Cancer Center, Tampere, Finland; 4 Finnish Cancer Registry, Helsinki, Finland; 5 Department of Urology, University of Helsinki and Helsinki University Hospital, Helsinki, Finland; 6 School of Health Sciences, Tampere University, Tampere, Finland; The University of Texas MD Anderson Cancer Center, UNITED STATES

## Abstract

The aim of this study was to investigate pre- and post-diagnostic use of antihypertensive drugs on prostate cancer (PCa)-specific survival and the initiation of androgen deprivation therapy (ADT). The cohort investigated 8,253 PCa patients with 837 PCa-specific deaths during the median follow-up of 7.6 years after diagnosis. Information on drug use, cancer incidence, clinical features of PCa, and causes of death was collected from Finnish registries. Hazard ratios with 95% confidence intervals were calculated using Cox regression with antihypertensive drug use as a time-dependent variable. Separate analyses were performed on PCa survival related to pre- and post-diagnostic use of drugs and on the initiation of ADT. Antihypertensive drug use overall was associated with an increased risk of PCa-specific death (Pre-PCa: 1.21 (1.04–1.4), Post-PCa: 1.2 (1.02–1.41)). With respect to the separate drug groups, angiotensin II type 1 receptor (ATr) blockers, were associated with improved survival (Post-PCa: 0.81 (0.67–0.99)) and diuretics with an increased risk (Post-PCa: 1.25 (1.05–1.49)). The risk of ADT initiation was slightly higher among antihypertensive drug users as compared to non-users. In conclusion, this study supports anti-cancer effect of ATr blockers on PCa prognosis and this should be investigated further in controlled clinical trials.

## Introduction

Prostate cancer (PCa) is the most common cancer in men [1]. Known risk factors for PCa are age, race, and a family history of PCa. Hypertension has been suggested as a risk factor for PCa progression [2–4]. However, hypertension is linked to other factors such as metabolic syndrome—thus, it is difficult to distinguish the impacts of underlying risk factors, hypertension and its medication on PCa development and progression. As in many other cancers, PCa involves re-programmed normal cellular functions, such as glucose- and cholesterol metabolism, in cancer progression [5]. Thus, commonly used drugs affecting normal cellular functions such as drugs targeting the renin-angiotensin system (RAS), fluid homeostasis, and the sympathetic nervous system may influence PCa progression.

In particular, the role of RAS in cancer development and progression has been under investigation [6, 7]. In mice, treatment with angiotensin II receptor blockers decreased the volume of a prostate tumor. However, administration of angiotensin II had no effect on tumor size [8]. On the other hand, angiotensin II has increased prostate cell viability in different cell models [9, 10].

Results on antihypertensive drug use and PCa-specific mortality are controversial [11–18]. In general, the evaluation of use of different antihypertensive drug groups is challenging due to the heterogeneity of drug users. Furthermore, differentially acting drug groups are commonly used in parallel to achieve blood pressure control.

Recently, we showed that the use of antihypertensive drugs is moderately associated with an increased risk for prostate cancer in a comprehensive population-based cohort study based on the Finnish Randomized Study of Screening for Prostate Cancer (FinRSPC). The risk increase was not related to any specific drug group [19]. Here we have investigated whether pre- and post-diagnostic use of any antihypertensive drug group associates with PCa-specific survival. Furthermore, we evaluated the risk of PCa progression by using initiation of androgen-deprivation therapy (ADT) as a surrogate.

## Materials and methods

### Study cohort

The original study cohort, FinRSPC, involved 80,458 men aged 55–67 years old at study entry from Tampere and Helsinki areas in Finland [20]. All men were free of PCa at baseline. By the end of 2016, 8,253 men were diagnosed with prostate cancer and information was available on their drug use. These men formed our study population. The Finnish Cancer Registry, which covers 96% of all solid tumor cases, provided information on cancer diagnoses [21]. Data on Gleason scores, TNM stage, and prostate cancer-specific antigen (PSA) level at the time of diagnosis was obtained from medical records. The Cause of Death registry in Statistics Finland houses information on causes of death (TK-53-1330-18) and the Care Register for Health Care provided information on diagnoses and medical procedures in secondary and tertiary health care units. Unique personal identification number, assigned to all Finnish citizens, was used in a deterministic linkage to combine information from the registries and medical records. Causes of death were classified using the International Classification of Diseases (ICD-10) codes; PCa was considered the cause of death when the underlying cause was C61 and deaths from cardiovascular disease (CVD) included codes I20-I25, I30-I52, and I70-I79. During 2004–2008, a questionnaire about height, weight, and use of non-prescription drugs, such aspirin and other non-steroidal anti-inflammatory drugs (NSAIDs) was mailed to participants still in the study. Originally, information on Body Mass Index (BMI) was collected from 11,698 subjects [22], and in the current study population, this was available for 805 men.

### Information on medication use

The prescription database of Social Insurance Institution (SII) of Finland was used to collect information on the use of antihypertensive drugs from 1996 to 2016. As a part of the national health insurance, SII provides reimbursements for purchases of physician-prescribed drugs to all Finnish citizens. All reimbursements are recorded in the database including the purchase date, drug dose, number of doses in the package, and number of drug packages for each purchase. In Finland, all antihypertensive drugs are available only by prescription, thus all purchases, except drugs for hospital patients, are recorded.

Drug-specific Anatomic Therapeutic Chemical (ATC) codes were used to identify the medications and were divided according to mechanism of action into angiotensin-converting enzyme (ACE) inhibitors, angiotensin II receptor (ATr) blockers, beta-blockers, calcium channel blockers, and diuretics. Additional information on purchases of statins, antidiabetic drugs, 5-alpha-reductase inhibitors, and prescription aspirin and other NSAIDs was also collected. The diuretics analyzed included only those drugs used for hypertension. Thus, loop-diuretics and spironolactone, commonly used for edema and fluid retention problems, were excluded.

Defined Daily Dose (DDD) [23] was used to calculate standardized cumulative doses by dividing annual amount of purchased drug by drug-specific DDD-values. All DDDs in each group were calculated together and value presented as annual cumulative doses of each drug group. The number of years with recorded drug purchases was used to calculate cumulative years of drug use. By dividing cumulative doses by cumulative years, average annual doses, i.e. average intensities of the drug use were calculated. Risk trends were analyzed by stratifying the study population by tertiles of intensity, DDD-values, and years of the drug use (termed low, medium and high use).

### Statistical analysis

Hazard ratios (HR) and their 95% confidence intervals (CI) for prostate cancer-specific mortality were estimated using Cox regression models. The time metric was months and years from diagnosis. Each antihypertensive drug group was analyzed as a time-dependent variable, except pre-diagnostic use which was analyzed as a time-fixed variable (thus subjects were classified as either ever-users or never-users before the diagnosis). Purchases were used to determine annually cumulative use for each follow-up year for each drug group. Subjects stayed as non-users until the first antihypertensive drug purchase and after that, they remained as ever-users for the whole follow-up period. This minimized bias due to selective discontinuation of medication in the terminal phase of cancer. Analysis of overall antihypertensive drug use was conducted separately. Different drug groups were included into models simultaneously as separate time-dependent variables, to enable modelling of mutually adjusted simultaneous use of several antihypertensive drugs. Lag-time analysis was conducted by lagging the diagnosis of PCa by one or three years from different drug use.

To evaluate whether antihypertensive drug use exerted any impact on the progression of cancer, the risk for initiation of androgen deprivation therapy (ADT) was analyzed using a Cox regression model, where follow-up started at PCa diagnosis and continued until ADT initiation, death/emigration, or the end of 2016. The analysis was limited only for long-term ADT treatment, participants who also had radiation therapy as primary management in addition to ADT were excluded.

Analyses were performed for risk overall and separately for subgroups of Gleason grade 7 and 8–10, risk group 2 (for definition see below), and metastatic cancers. The risk for all-cause mortality was calculated without subgroup analyses. All analyses were adjusted for age, FinRSPC trial arm (screening arm and control arm), year of diagnosis, cancer clinical characteristics (T stage, metastasis, and Gleason grade), Charlson comorbidity index, use of statins, antidiabetic drugs, anticoagulants, 5-alpha-reductase inhibitors, aspirin and other NSAIDs. Sensitivity analysis by adjusting the model by marital and socioeconomical status, and BMI were conducted with subgroup in whom information was available. We also conducted sensitivity analysis by number of used drugs (one, two, or three or more) and combination of different drug groups (e.g. ATr blockers + diuretics). PCa risk groups were created based on Gleason grade, clinical characteristics and tumor extent: risk group 0 included cases where Gleason grade was 6, T-stage was 1 or 2, or PSA was less than 10 μg/l, risk group 1 included cases where Gleason grade was 7, T-stage was 3, or PSA was 10–20 μg/l and risk group 2 included cases where Gleason grade was 8 or more, T-stage was 4, cancer was metastatic, or PSA was more than 20 μg/l. Charlson comorbidity index was calculated as explained previously [24]. All analyses were performed using IBM SPSS statistical software (version 24).

We performed competing risks analysis to estimate association between antihypertensive drug groups and PCa-specific death, with deaths from cardiovascular disease (CVD) as the competing risk. Deaths due to non-cancer and non-CVD causes were censored. The model was adjusted for age, FinRSPC trial arm, year of diagnosis, PCa risk group, Charlson comorbidity index, use of statins, antidiabetic drugs, anticoagulants, 5-alpha-reductase inhibitors, aspirin, and other NSAIDs. The analysis was done using StataCorp Stata Statistics (version 14.0).

## Results

### Population characteristics

The majority (79%) of the study population had at least one antihypertensive drug purchase during the follow-up (Table 1). Of the 8,253 prostate cancer cases, 2,479 had Gleason grade 7 and 1,379 Gleason 8 or above, 2284 patients belonged to risk group 2, and 589 had metastatic cancer. The median follow-up time was 7.6 years after diagnosis. The median age at diagnosis was 68 years. In total, 2,765 subjects died during the follow-up, including 837 deaths from PCa (Table 1). The overall PCa-specific death rate after diagnosis was 13.3 per 1000 person-years. It was 12.0 among users and 20.2 in non-users (Table 1). Of the specific drug groups, PCa-specific mortality after diagnosis was highest among users of diuretics (11.7 deaths per 1000 person-years) and lowest among users of ATr blockers (8 per 1000) (Table 1).

**Table 1:** Population characteristics. Cohort of 8253 men with prostate cancer (PCa) from the Finnish Randomized Study of Screening for Prostate Cancer. ACE inhibitors = angiotensin-converting enzyme inhibitors; ATr blockers = angiotensin II receptor type.

	All	Non-users	Users	ACE inbitors	ATr blockers	Beta-blockers	Calcium channel blockers	Diuretics
NO. of PCa cases	8253	1875 (22.7)	6378 (77.3)	3297 (39.9)	2316 (28.1)	4467 (54.1)	3172 (38.4)	2397 (29)
Gleason 7 n (%)	2479 (30)	539 (28.7)	1940 (30.5)	990 (30.0)	694 (30.0)	1355 (30.3)	958 (30.2)	705 (29.4)
Gleason 8–10 n (%)	1379 (16.7)	345 (18.4)	1034 (16.2)	510 (15.5)	352 (15.2)	705 (15.8)	464 (14.6)	361 (15.1)
Risk group 2 n (%)	2284 (27.7)	559 (29.8)	1725 (27)	877 (26.6)	560 (24.2)	1211 (27.1)	811 (25.6)	627 (26.2)
Metastatic ceses n (%)	589 (7.1)	180 (9.6)	409 (6.4)	208 (6.3)	99 (4.3)	277 (6.2)	173 (5.5)	141 (5.9)
Hormonal therapy n (%) (excluded cases where radiation therapy was combined with ADT)	2120 (25.7)	483 (25.8)	1637 (25.7)	872 (26.4)	469 (20.3)	1149 (25.7)	764 (24.1)	581 (24.2)
Median age at time of randomization (IQR)	63 (59–63)	59 (55–63)	63 (59–67)	63 (59–67)	59 (59–63)	63 (59–67)	63 (59–67)	63 (59–67)
Median age at time of diagnosis (IQR)	68 (64–72)	68 (64–72)	69 (65–72)	68 (64–72)	68 (64–72)	69 (65–73)	68 (65–72)	68 (64–72)
Follow-up time after randomization, median (IQR)	12 (11–13)	12 (11–13)	12 (11–13)	12 (11–13)	12 (11–13.4)	12 (11–13)	12 (11–13)	12 (11–13)
Follow-up time after diagnosis, median (IQR)	7.6 (3.8–11.1)	5.9 (2.8–10.3)	8 (4.2–11.3)	8.4 (4.6–11.8)	8.7 (4.8–11.8)	8.1 (4.3–11.5)	8.5 (4.7–11.7)	8.9 (5–12)
Deaths n (%)	2765 (33.5)	622 (33.2)	2143 (33.6)	1148 (34.8)	663 (28.6)	1587 (35.5)	1042 (32.8)	805 (33.6)
PCa-specifin deaths n (% from all deaths)	837 (30.2)	224 (36)	613 (28.6)	308 (26.8)	162 (24.4)	415 (26.1)	271 (26)	249 (30.9)
PCa-specifin deaths per 1000 person-years after diagnosis	13.3	20.2	12.0	11.1	8.0	11.5	10.1	11.7
CVD-specific deaths n (% from all deaths)	586 (21.2)	66 (10.6)	520 (8.2)	328 (28.6)	175 (26.4)	420 (26.5)	259 (24.9)	197 (24.4)
Median BMI (IQR) (n)	26.24 (24.22–28.67) (805)	25.06 (23.54–27.17) (197)	26.59 (24.55–28.73) (608)***	26.6 (24.4–29.0) (277)***	27.1 (25.0–29.1) (242)***	27.0 (24.9–29.1) (391)***	26.9 (24.9–29.1) (299)***	27.74 (25.42–30.05) (202)
**FinRSPC arm**								
Screening n (%)	3425 (41.5)	797 (42.5)	2628 (41.2)	1391 (42.2)	934 (40.3)	1855 (41.5)	1318 (41.6)	962 (40.1)
Control n (%)	4828 (58.5)	1078 (57.5)	3750 (58.8)	1906 (57.8)	1382 (59.7)	2612 (58.5)	1854 (58.4)	1435 (59.9)
**Use of other drugs**								
Statins n (%)	3870 (46.9)	408 (21.8)	3462 (54.3)***	1913 (58.0)***	1383 (59.7)***	2604 (58.3)***	1825 (57.5)***	1382 (57.7)***
Antidiabetic drugs n (%)	1599 (19.4)	118 (6.3)	1481 (23.3)***	915 (27.8)***	608 (26.2)***	1130 (25.3)***	853 (26.9)***	617 (25.7)***
NSAIDs n (%)	7065 (85.6)	1534 (81.8)	5531 (86.7)***	2821 (85.6)***	2058 (88.9)***	3881 (86.9)***	2771 (87.4)***	2107 (87.9)***
Aspirin n (%)	1143 (13.8)	139 (7.4)	1004 (15.7)***	522 (15.8)***	404 (17.4)***	760 (17.0)***	528 (16.6)***	374 (15.6)***
5-alpha-reductase inhibitors n (%)	1229 (14.9)	224 (11.9)	1005 (15.8)***	495 (15.0)***	381 (16.5)***	717 (16.1)***	499 (15.7)***	390 (16.3)***
Anticoagulants n (%)	4275 (51.8)	585 (31.2)	3690 (57.9)***	2050 (62.2)***	1370 (59.2)***	2895 (64.8)***	1867 (58.9)***	1388 (57.9)***

The users of antihypertensive drugs also had been prescribed other drugs more often than non-users (p<0.001) (Table 1). Non-users had slightly lower BMI values. There was no difference in the distribution of FinRSPC study arms or age at diagnosis between the groups (68 or 69 years in all groups). The follow-up time was slightly shorter in the group of non-users, however, it was similar between all other groups (Table 1).

### Risk of prostate cancer-specific death by antihypertensive drug use

**Fig 1:**
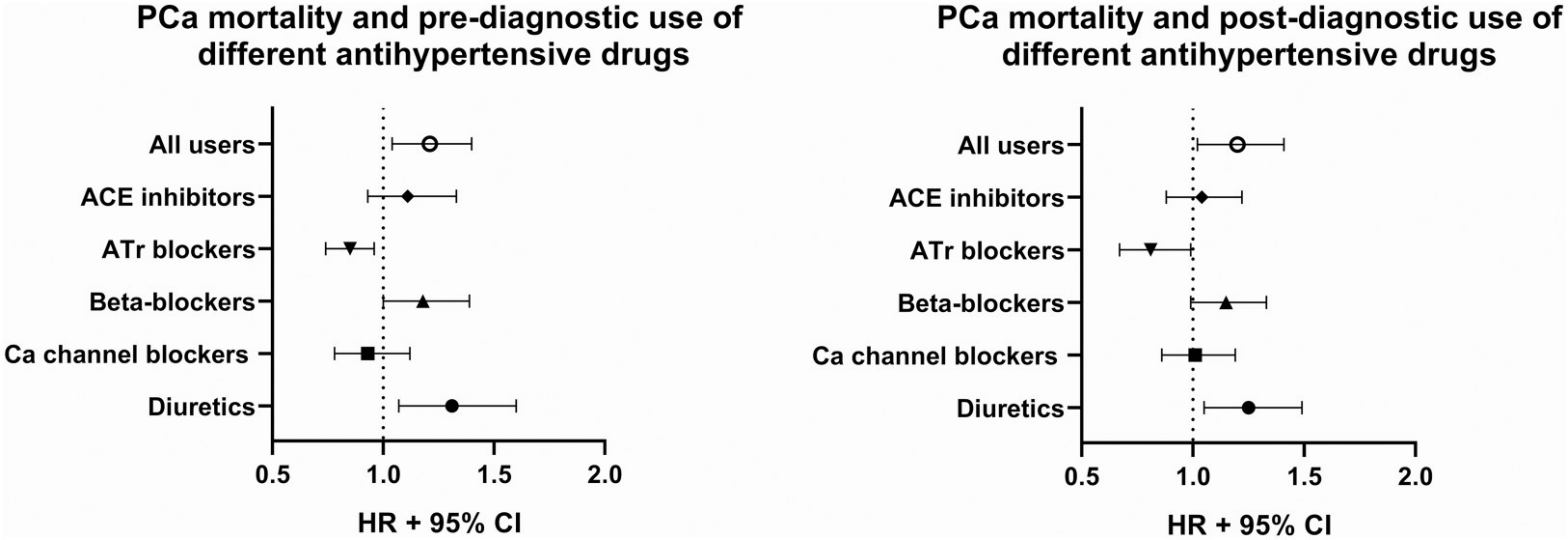
Association of prostate cancer (PCa)-specific mortality and pre- and post-diagnostic use of different antihypertensive drugs. Data is presented as hazard ratios (HR) with 95% confidence intervals (CI).

Both pre- and post-diagnostic use of antihypertensive drugs was associated with an increased risk for PCa-specific death compared to non-users in multivariable-adjusted analysis (HR 1.21, 95% CI 1.04–1.4 and HR 1.2, 95% CI 1.02–1.41, respectively) (Fig 1, Table 2). In addition, the risk for all-cause mortality was higher among users of antihypertensive drugs (Pre: HR 1.38, 95% CI 1.27–1.5, Post: HR 1.33, 95% CI 1.21–1.47) (Table 3A). As expected, the risk of CVD-specific deaths was elevated in users of antihypertensive drugs (Pre: HR 1.96 95% CI 1.61–2.39, Post: HR 2.57, 95% CI 1.97–3.36) (Table 3B).

**Table 2:** Risk for prostate cancer (PCa)-specific mortality of pre- and post-diagnostic use of antihypertensive drugs compared to non-users after PCa diagnosis. Cox regression hazard model was adjusted with age, FinRSPC trial arm (screening arm and control arm), year of diagnosis, cancer clinical characteristics (T stage, metastasis, and Gleason grade), Charlson comorbidity index, use of statins, antidiabetic drugs, anticoagulants, 5-alpha-reductase inhibitors, aspirin and other NSAIDs. ACE inhibitors = angiotensin-converting enzyme inhibitors; ATr blockers = angiotensin II receptor type 1 blockers; HR (95% CI) = hazard ratio and 95% confidence intervals.

PCa-specific death			Pre-diagnostic use	Post-diagnostic use
Overall risk	n of men	n of PCa deaths (%)	HR (95% CI)	HR (95% CI)
Non-users	1875	224 (11.9)	ref.	ref.
Users	6378	613 (9.6)	1.21 (1.04–1.4)	1.2 (1.02–1.41)
ACE inhibitors	3297	308 (9.3)	1.11 (0.93–1.33)	1.04 (0.88–1.22)
ATr blockers	2316	162 (7)	0.74 (0.58–0.96)	0.81 (0.67–0.99)
Beta-blockers	4467	415 (9.3)	1.18 (1.0–1.39)	1.15 (0.99–1.33)
Calcium channel blockers	3172	271 (8.5)	0.93 (0.78–1.12)	1.01 (0.86–1.19)
Diuretics	2397	249 (10.4)	1.31 (1.07–1.6)	1.25 (1.05–1.49)
**Gleason 7**				
Non-users	539	50	ref.	ref.
Users	1940	178	1.69 (1.28–2.26)	1.39 (1–1.94)
ACE inhibitors	1489	130	1.26 (0.89–1.78)	1.26 (0.97–1.76)
ATr blockers	1785	179	0.89 (0.56–1.43)	0.76 (0.53–1.09)
Beta-blockers	1124	111	1.31 (0.96–1.8)	1.07 (0.79–1.44)
Calcium channel blockers	1521	141	0.86 (0.6–1.23)	1.05 (0.78–1.41)
Diuretics	1774	145	1.48 (1.1–2.16)	1.55 (1.13–2.15)
**Gleason 8–10**				
Non-users	345	115	ref.	ref.
Users	1034	288	1.05 (0.84–1.31)	1.11 (0.88–1.41)
ACE inhibitors	510	134	1.1 (0.85–1.42)	0.91 (0.72–1.15)
ATr blockers	352	81	0.72 (0.51–1.02)	0.9 (0.68–1.2)
Beta-blockers	705	195	1.14 (0.9–1.45)	1.12 (0.96–1.48)
Calcium channel blockers	464	119	0.98 (0.76–1.26)	1.01 (0.8–1.28)
Diuretics	361	103	1.24 (0.94–1.65)	1.08 (0.8–1.28)
**Risk group 2**				
Non-users	559	179	ref.	ref.
Users	1725	426	1.12 (0.94–1.33)	1.1 (0.91–1.33)
ACE inhibitors	1407	214	1.18 (0.95–1.45)	1.1 (0.91–1.33)
ATr blockers	1724	106	0.7 (0.52–0.94)	0.82 (0.65–1.05)
Beta-blockers	1073	282	1.13 (0.94–1.38)	1.08 (0.9–1.23)
Calcium channel blockers	1473	181	0.93 (0.76–1.15)	0.99 (0.82–1.2)
Diuretics	1657	163	1.31 (1.03–1.66)	1.15 (0.93–1.42)
**Metastatic disease**				
Non-users	180	112	ref.	ref.
Users	409	213	1.04 (0.82–1.32)	0.95 (0.74–1.22)
ACE inhibitors	381	106	1.38 (1.02–1.85)	1.1 (0.83–1.45)
ATr blockers	490	41	0.73 (0.48–1.12)	0.83 (0.57–1.22)
Beta-blockers	312	131	0.95 (0.73–1.24)	0.93 (0.73–1.19)
Calcium channel blockers	416	87	1 (0.75–1.34)	1.08 (0.82–1.42)
Diuretics	448	67	1.01 (0.73–1.41)	0.89 (0.64–1.23)

**Table 3:** Risk for all-cause mortality (A), and cardiovascular diseases (CVD) mortality of pre- and post-diagnostic use of antihypertensive drugs compared to non-users after PCa diagnosis. Cox regression hazard model was adjusted with age, FinRSPC trial arm (screening arm and control arm), year of diagnosis, cancer clinical characteristics (T stage, metastasis, and Gleason grade), Charlson comorbidity index, use of statins, antidiabetic drugs, anticoagulants, 5-alpha-reductase inhibitors, aspirin and other NSAIDs. ACE inhibitors = angiotensin-converting enzyme inhibitors; ATr blockers = angiotensin II receptor type 1 blockers; HR (95% CI) = hazard ratio and 95% confidence intervals.

**A)**				
**All-cause death**			**Pre-diagnostic use**	**Post-diagnostic use**
**Overall risk**	n of men	n of deaths	HR (95% CI)	HR (95% CI)
Non-users	1875	622	ref.	ref.
Users	6378	2143	1.38 (1.27–1.5)	1.33 (1.21–1.47)
ACE inhibitors	3297	1148	1.22 (1.11–1.34)	1.19 (1.09–1.29)
ATr blockers	2316	663	0.88 (0.77–1)	0.98 (0.89–1.08)
Beta-blockers	4467	1587	1.22 (1.12–1.33)	1.4 (1.28–1.52)
Calcium channel blockers	3172	1024	1.05 (0.96–1.16)	1.07 (0.99–1.17)
Diuretics	2397	805	1.16 (1.05–1.29)	0.97 (0.88–1.07)
**B)**				
**CVD death**			**Pre-diagnostic use**	**Post-diagnostic use**
**Overall risk**	n of men	n of CVD deaths	HR (95% CI)	HR (95% CI)
Non-users	1875	66	ref.	ref.
Users	6378	520	1.96 (1.61–2.39)	2.57 (1.97–3.36)
ACE inhibitors	3297	328	1.52 (1.25–1.85)	1.85 (1.54–2.22)
ATr blockers	2316	175	0.83 (0.64–1.08)	1.26 (1.03–1.54)
Beta-blockers	4467	420	1.67 (1.39–2.02)	2.07 (1.7–2.53)
Calcium channel blockers	3172	259	1.12 (0.92–1.36)	0.88 (0.72–1.06)
Diuretics	2397	197	1.11 (0.9–1.37)	1.13 (0.95–1.36)

**Fig 2:**
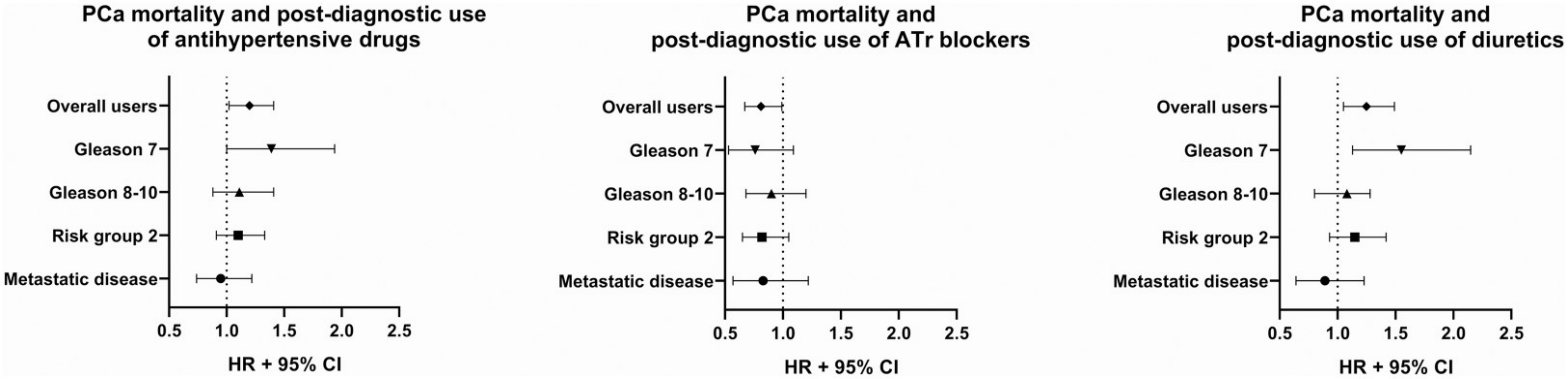
Role of tumor clinical characteristics on PCa mortality among users of any antihypertensive drug, and separately for angiotensin II receptor blockers (ATr blockers) and diuretics. Data is presented as hazard ratios (HR) with 95% confidence intervals (CI).

When antihypertensive drug groups were analyzed separately, post-diagnostic use of ATr blockers was associated with a decreased risk for PCa death (HR 0.81, 95% CI 0.67–0.99) (Fig 1, Table 2). The risk decrease was observed also for pre-diagnostic use (HR 0.74, 95% CI 0.58–0.96). Pre- and post-diagnostic use of diuretics was associated with an increased risk of death from PCa (Pre: HR 1.31, 95% CI 1.07–1.6, Post: HR 1.25, 95% CI 1.05–1.49) (Fig 1, Table 2). Tumor characteristics did not modify the risk associations by the use of ATr blockers or diuretics (Fig 2, Table 2).

Furthermore, pre-diagnostic use of ATr blockers was associated with a decreased all-cause mortality (Table 3A). On the other hand, the use of beta-blockers and ACE inhibitors was associated with increased all-cause mortality (Table 3A). Other investigated antihypertensive drug group did not associate with the risk of PCa-specific or all-cause mortality (Fig 1, Tables 2 & 3A). The use of beta-blockers and ACE inhibitors also associated with elevated CVD mortality (Table 3B). Diuretics and calcium channel blockers displayed no association with deaths from CVD.

Adjustments of the model by socioeconomical and marital status, and BMI did not modify the risk associations. Lagging the PCa diagnosis by one or three years after drug use did not reveal any statistically meaningful association with prostate cancer-specific mortality (S3 Table).

Sensitivity analysis by number of used drugs did not modify the results as risk for PCa-specific death was increased similarly despite number of drug groups in use (S5 Table). Some drug combinations showed slightly different survival associations compared to main analysis; combination of beta-blockers and diuretics were associated with lowered risk of PCa death compared non-users (HR 0.74, 95% CI 0.57–0.96) whereas diuretics and beta-blockers in combination were associated with increased risk (HR 2.36, 95% CI 1.89–2.94). Other drug group combination did not show significant associations with PCa-specific survival (S5 Table).

### Risk trends by cumulative antihypertensive drug use

In the risk trend analysis for the post-diagnostic use of ACE inhibitors, the risk for PCa death tended to decrease with the amount of use, but the trend was not statistically significant (Table 4). Among ATr blocker users, the strongest risk decrease was observed in the tertile of highest intensity of use (over 559.2 DDDs annually) regardless of cancer grade and stage (Table 4). No clear dose-dependent risk trends were observed by pre-diagnostic use for either drug group (S1 Table). Risk trend analyses for tertiles of post-diagnostic DDD-values and years of use can be found in the supplemental material (S2 Table).

**Table 4:** Risk of prostate cancer (PCa)-specific mortality by the post-diagnostic use of antihypertensive drugs after diagnosis of PCa. Users stratified into tertiles by cumulative intensity of the use (DDD values/years of the use). Cox regression model was adjusted with age, FinRSPC trial arm (screening arm and control arm), year of diagnosis, cancer clinical characteristics (T stage, metastasis, and Gleason grade), Charlson comorbidity index, use of statins, antidiabetic drugs, anticoagulants, 5-alpha-reductase inhibitors, aspirin and other NSAIDs. ACE inhibitors = angiotensin-converting enzyme inhibitors; ATr blockers = angiotensin II receptor type 1 blockers; HR (95% CI) = hazard ratio with 95% confidence intervals.

	ACE inhibitors	ATr blockers	Beta-blockers	Calcium channel blockers	Diuretics
**Limits**					
Low	<336	<303.2	<100	<261.3	<148.3
Medium	336–676.9	303.2–559.2	100–187.4	261.3–399.4	148.3–217.8
High	676.9>	559.2>	187.4>	399.4>	217.8>
**Overall, n**					
Low	1100	772	1542	1051	790
Medium	1098	772	1436	1064	790
High	1099	772	1489	1057	790
**Overall, PCa death risk**	**HR (95% CI)**	**HR (95% CI)**	**HR (95% CI)**	**HR (95% CI)**	**HR (95% CI)**
Low	1.16 (0.93–1.45)	0.89 (0.67–1.18)	1.27 (1.04–1.55)	1.18 (0.94–1.48)	1.44 (1.12–1.84)
Medium	1.06 (0.84–1.33)	0.81 (0.6–1.09)	1.29 (1.05–1.6)	0.93 (0.73–1.19)	1.09 (0.82–1.45)
High	0.92 (0.72–1.19)	0.63 (0.45–0.88)	0.97 (0.78–1.21)	1.08 (0.84–1.4)	1.32 (1.02–1.7)
**Gleason 7, n**					
Low	302	201	465	379	240
Medium	337	255	228	408	215
High	351	238	462	315	241
**Gleason 7, PCa death risk**	**HR (95% CI)**	**HR (95% CI)**	**HR (95% CI)**	**HR (95% CI)**	**HR (95% CI)**
Low	1.15 (0.75–1.78)	0.99 (0.6–1.64)	1.04 (0.71–1.55)	1.11 (0.74–1.65)	1.92 (1.27–2.91)
Medium	1.28 (0.84–1.96)	0.76 (0.44–1.33)	0.92 (0.6–1.42)	1.15 (0.75–1.75)	1.3 (0.78–2.15)
High	1.67 (1.09–2.57)	0.69 (0.37–1.26)	1.17 (0.78–1.74)	0.92 (0.55–1.55)	1.28 (0.79–2.08)
**Gleason 8–10, n**					
Low	168	134	256	288	104
Mediate	178	102	229	226	132
High	164	116	220	116	119
**Gleason 8–10, PCa death risk**	**HR (95% CI)**	**HR (95% CI)**	**HR (95% CI)**	**HR (95% CI)**	**HR (95% CI)**
Low	1.06 (0.75–1.48)	0.94 (0.63–1.41)	1.58 (1.18–2.12)	1.38 (0.97–1.97)	1.21 (0.81–1.81)
Medium	0.89 (0.63–1.25)	0.85 (0.55–1.31)	1.5 (1.11–2.02)	0.95 (0.65–1.38)	1.02 (0.67–1.55)
High	0.69 (0.47–1.02)	0.58 (0.36–0.95)	0.85 (0.61–1.2)	1.15 (0.81–1.65)	1.24 (0.84–1.83)
**Risk group 2, n**					
Low	287	201	417	476	207
Medium	301	183	394	369	220
High	289	176	400	231	193
**Risk group 2, PCa death risk**	**HR (95% CI)**	**HR (95% CI)**	**HR (95% CI)**	**HR (95% CI)**	**HR (95% CI)**
Low	1.23 (0.94–1.61)	0.9 (0.64–1.27)	1.28 (1–1.62)	1.17 (0.89–1.54)	1.27 (0.93–1.74)
Medium	1.06 (0.81–1.41)	0.77 (0.53–1.13)	1.25 (0.97–1.62)	0.87 (0.64–1.18)	1.09 (0.78–1.53)
High	1.01 (0.75–1.35)	0.62 (0.41–0.94)	0.91 (0.7–1.19)	1.15 (0.85–1.55)	1.25 (0.91–1.71)
**Metastatic cancer, n**					
Low	77	33	104	154	43
Medium	69	39	91	78	47
High	62	27	82	27	48
**Metastatic cancer, PCa death risk**	**HR (95% CI)**	**HR (95% CI)**	**HR (95% CI)**	**HR (95% CI)**	**HR (95% CI)**
Low	1.26 (0.86–1.85)	0.91 (0.52–1.61)	1.02 (0.72–1.44)	1.37 (0.93–2.03)	0.79 (0.47–1.33)
Medium	1.24 (0.84–1.83)	0.9 (0.52–1.56)	1.4 (0.98–2)	0.75 (0.46–1.21)	0.83 (0.5–1.38)
High	1.02 (0.67–1.55)	0.42 (0.2–0.88)	0.62 (0.42–0.91)	1.55 (1.02–2.33)	1.29 (0.81–2.07)

### Risk for initiation of androgen deprivation therapy

Any use of antihypertensive drugs was associated with a slightly increased risk for initiation of ADT as compared to non-users (HR 1.15, 95% CI 1.05–1.27) (Table 4). The risk increase was seen also in Gleason 8–10 and risk group 2 tumors (Table 5). When different drug groups were compared, only use of ACE inhibitors and beta blockers showed increased risks (HR 1.21, 95% CI 1.09–1.35, and HR 1.1, 95% CI 1–1.21, respectively). Such associations were not seen in the subgroups of clinical tumor characteristics (Table 5). Drug groups did not reveal any meaningful dose-dependent association with the risk of initiation of ADT (S4 Table).

**Table 5:** Effect of antihypertensive drugs on initiation of hormonal therapy for treatment of prostate cancer in Finnish men. Cox regression hazard model was adjusted with age, FinRSPC trial arm (screening arm and control arm), year of diagnosis, cancer clinical characteristics (T stage, metastasis, and Gleason grade), Charlson comorbidity index, use of statins, antidiabetic drugs, anticoagulants, 5-alpha-reductase inhibitors, aspirin and other NSAIDs. ADT = androgen deprivation therapy; ACE inhibitors = angiotensin-converting enzyme inhibitors; ATr blockers = angiotensin II type 1 receptor blockers; HR (95% CI) = hazard ratio with 95% confidence intervals.

		Initiation of ADT
Overall risk	n of ADT treated men	HR (95% CI)
Non-users	483	ref.
Users	1637	1.15 (1.05–1.27)
ACE inhibitors	872	1.21 (1.09–1.35)
ATr blockers	469	1.05 (0.91–1.21)
Beta-blockers	1149	1.1 (1-1-21)
Calcium channel blockers	764	0.86 (0.76–0.98)
Diuretics	581	1 (0.89–1.11)
**Gleason 7**		
Non-users	298	ref.
Users	958	1.13 (0.94–1.36)
ACE inhibitors	270	1.16 (0.95–1.41)
ATr blockers	141	1.07 (0.83–1.39)
Beta-blockers	356	1.12 (0.94–1.34)
Calcium channel blockers	243	1.05 (0.86–1.29)
Diuretics	335	0.8 (0.62–1.02)
**Gleason 8–10**		
Non-users	219	ref.
Users	548	1.2 (1–1.43)
ACE inhibitors	231	1.22 (0.99–1.5)
ATr blockers	129	1.11 (0.84–1.46)
Beta-blockers	319	1.09 (0.91–1.31)
Calcium channel blockers	205	099 (0.81–1.23)
Diuretics	184	0.92 (0.81–1.23)
**Risk group 2**		
Non-users	360	ref.
Users	952	1.15 (1.01–1.32)
ACE inhibitors	415	1.19 (1.02–1.39)
ATr blockers	208	1.1(0.89–1.36)
Beta-blockers	559	1.04 (0.91–1.2)
Calcium channel blockers	369	0.97 (0.83–1.14)
Diuretics	336	0.91 (0.75–1.1)
**Metastatic disease**		
Non-users	166	ref.
Users	381	1.08 (0.88–1.31)
ACE inhibitors	189	1.09 (0.87–1.38)
ATr blockers	87	0.94 (0.66–1.33)
Beta-blockers	246	1 (0.82–1.22)
Calcium channel blockers	158	1.03 (0.81–1.3)
Diuretics	136	0.96 (0.73–1.27)

### Competing risk analysis

The risk association for the pre-diagnostic use antihypertensive drug use was similar in competing risk analysis as in the main analysis; use of ATr blockers continued to be associated with improved disease-specific survival (HR 0.74, 95% CI 0.57–0.98) and the use of diuretics slightly increased the risk (HR 1.25, 95% CI 1.01–1.54).

## Discussion

We evaluated prostate cancer-specific and all-cause mortality by pre- and post-diagnostic antihypertensive drug use in a Finnish cohort study consisting of men living in metropolitan areas of Helsinki and Tampere. In general, the use of antihypertensive drugs was associated with increased PCa-specific and all-cause mortality as compared to non-users. When we evaluated the different antihypertensive drug groups, post-diagnostic use of RAS-inhibiting drugs, ACE inhibitors, and AT-receptor blockers were associated with improved survival, whereas diuretics were associated with poorer survival.

The reduction in risk was more pronounced for AT-receptor blockers than for ACE inhibitors. However, only post-diagnostic use of ACE inhibitors showed a dose-dependent risk trend. Similarly, in a UK population-based cohort, Cardwell et al. [13] revealed that users of ACE inhibitors and ATr blockers had a slightly decreased PCa-specific mortality and concluded that it was safe to use these antihypertensive drugs after PCa diagnosis. Furthermore, Ronquist et al. [25] observed that the post-operative captopril users had less biochemical failures as compared to non-users. However, the study consisted only of 62 patients and the mean follow-up time was 29 months. Alashkham et al. [26] showed that biochemical recurrence was decreased after curative-intent radiotherapy with hormone treatment among users of RAS-inhibiting drugs as compared to non-users. Our study supports these findings, although we did not see any reduction in the risk of initiation of ADT therapy among users of RAS-inhibiting drugs.

Results from analysis of pre-diagnostic use of antihypertensive drugs and PCa-specific survival was in line with the results of post-diagnostic use: overall antihypertensive drug use was associated with an increased risk for PCa-specific death, with decreased mortality among users of ATr blockers and an increase in users of diuretics. Furthermore, pre-diagnostic use of antihypertensive drugs was associated with increased all-cause mortality. This was also seen with several different drug groups: ACE inhibitors, beta-blockers, and calcium channel blockers, but not simply ATr blockers. These results underline that, in general, users of antihypertensive drugs are at an increased risk of death as compared to non-users, but the association is different for users of ATr blockers. A similar trend was evident when the association of antihypertensive drugs and CVD-specific deaths was analyzed. The results were similar when deaths due to CVD were taken into account using competing risk analysis, suggesting that the risk association with PCa survival was not affected by the higher risk for CVD death among antihypertensive drug users compared to non-users.

The reason why specifically it is the use of ATr blockers that may improve PCa-specific survival is not clear. In our study, users of ATr blockers had a smaller proportion of metastatic cancer cases, a lower likelihood of initiation of ADT therapy, and lower PCa mortality as compared to patients in the other groups. This could be due to selective prescribing of this drug group to healthier men than other antihypertensive drugs, or due to genuine effects of ATr blockers on PCa. Our results are in line with a recently published study, where the use of ATr blockers increased PCa-specific survival after radical prostatectomy [18]. ATr blockers are antagonists of angiotensin II receptor type 1, which mediates the classical angiotensin II-related effects; vasoconstriction, fluid volume homeostasis, cell proliferation, and fibrosis [27]. Other RAS-inhibiting drugs, ACE inhibitors, inhibit the main angiotensin II forming enzyme, thus both drug groups block angiotensin II-mediated functions, albeit at different sites in the pathway. ATr blockers may have some yet unknown mechanism of action impacting on PCa development and progression. Nevertheless, the possible role of RAS in development and progression of cancer is under investigation [7,28,29].

Diuretics are commonly used in management of edema, which is common in advanced cancer. Thus, loop diuretics (furosemide) and spironolactone, which are used in management of edema rather than hypertension, were excluded from this analysis. Still, the use of diuretics was associated with an increased risk for PCa death despite lack of association with a higher stage cancer, such metastatic or risk group 2 cases, which might be expected if increased the association could be explained by the treatment of end-stage cancer-related problems. In agreement, Holmes et al. [11] reported an increased risk for PCa-specific deaths among thiazide diuretics users. In our study, we did not evaluate the role of separate diuretic classes.

We did not find any association of pre- and post-diagnostic use of beta-blockers with prostate cancer-specific mortality. Previous studies have reported controversial results [14–17]. However, in analyses based on time-dependent variables, as in our study, no association has been found [30, 31]. However, the use of beta-blockers was associated with a slightly increased risk for ADT initiation, pointing to some association with tumor progression. In contrast to our findings, in a Norwegian population-based cohort, Grytli et al. [16] showed that the use of beta-blockers during ADT-therapy seemed to decrease the risk of PCa-specific death. However, that study was analyzed without time-dependent variables. Nevertheless, the link between beta-blockers and PCa outcomes remains unclear.

A recently published study [32] investigated the role of antihypertensive drug use on PCa-specific mortality in gonadotropin-releasing hormone agonist users. They showed a slightly increased PCa mortality in users of blood pressure lowering drugs. We did not have information on blood pressure levels and were thus unable to evaluate the role of hypertension in this risk association.

Our study has several strengths. The Finnish prescription database is comprehensive and accurate; for example, all reimbursements for purchases of antihypertensive drugs are recorded, and thus we had nearly complete coverage of our subjects’ drug purchases. Our follow-up time was long enough to enable modeling cumulative simultaneous use of multiple drugs. We also had comprehensive information of clinical features of prostate cancer, such as Gleason grade and TNM stage. In addition, information on PCa deaths was collected from an accurate and reliable nationwide, population-based database that has been validated by an independent cause of death committee; the validity of information of PCa-specific deaths between cause of death registry and recording in patients’ medical files was compared and 97.7% agreement (kappa = 0.95) between them was found [33]. Furthermore, statistical power in our cohort is sufficient to detect clinically meaningful survival differences as our sample size was sufficient to detect 3.5% survival difference between medication users and non-users with 80% power and risk for type I error being 0.05.

Our study also has some limitations. We did not have information on blood pressure levels of the subjects or indication for antihypertensive drugs. In addition, data of some background information, which might be shared risk factors for prostate cancer death and hypertension, such as smoking habits, were missing. However, we were able to adjust the analysis for the use of other drugs which might have impacted on the development and progression of PCa, such as statins [34]. Our drug use data is based on reimbursement for drug purchases. Thus, we do not know whether the subject actually used the purchased drugs. It should be noted that most likely the users of antihypertensive drugs have more comorbidities and are at increased risk of death in general as compared to non-users, possibly affecting also PCa-specific mortality. Furthermore, different antihypertensive drugs are described based on comorbidities and the health status of the patients which might bias the results.

In conclusion, the use of antihypertensive drugs was associated with an increased risk for prostate cancer-specific and all-cause mortality. When the analysis was done separately for different drug groups, both pre- and post- diagnostic use of RAS inhibiting drugs, especially ATr blockers, associated with improved PCa survival in a dose-dependent manner, whereas the use of diuretics associated with poorer survival. The results might be affected by systematic differences between the users and non-users. Nevertheless, our findings support previous reports about the beneficial effects of RAS-inhibition on PCa progression. The role and potential benefits of RAS inhibition in PCa should be examined further.

## Supporting information

S1 Table Risk for pre-diagnostic use of antihypertensive use on prostate cancer-specific mortality. Users stratified by intensity of use into tertiles (low, medium and high dose). ACE inhibitors = angiotensin-converting enzyme inhibitors and Atr blockers = angiotensin II type 1 receptor blockers.
(XLSX)

S2 Table Risk of prostate cancer-specific mortality according to the use of antihypertensive drugs after diagnosis of PCa. Users stratified to tertiles by cumulative years of the use (A) and cumulative DDD-values (B). ACE inhibitors = angiotensin-converting enzyme inhibitors, ATr blockers = angiotensin II type 1 receptor blockers, HR (95% CI) = hazard ratio with 95% confidence interval.
(XLSX)

S3 Table Lagtime analysis using 1-year and 3-year lagtimes of use of different antihypertensive drugs on PCa-specific mortality. Cox regression model was adjusted with age, FinRSPC trial arm (screening arm and control arm), year of diagnosis, cancer clinical characteristics (T stage, metastasis, and Gleason grade), Charlson comorbidity index, use of statins, antidiabetic drugs, anticoagulants, 5-alpha-reductase inhibitors, aspirin and other NSAIDs.
(XLSX)

S4 Table Risk of initiate hormonal treatment for prostate cancer by the use of antihypertensive drugs, users stratified by cumulative intensity of the use (DDD values/years of the use). ACE inhibitors = angiotensin-converting enzyme inhibitor, ATr = angiotensin II type 1 receptor blockers.
(XLSX)

S5 Table Sensitivity analysis of number of used drugs and combination of different drug groups on PCa-specific death. (XLSX)

## References

[1] Finnish cancer registry statistics. http://stats.cancerregistry.fi

[2] De Nunzio C, Aronson W, Freedland SJ, Giovannucci E, Parsons JK. The correlation between metabolic syndrome and prostatic diseases. Eur Urol. 2012;61: 560–570. 10.1016/j.eururo.2011.11.013 22119157

[3] Liang Z, Xie B, Li J, Wang X, Wang S, Meng S, et al Hypertension and risk of prostate cancer: a systematic review and meta-analysis. Sci Rep. 2016;6: 31358 10.1038/srep31358 27511796 PMC4980763

[4] Arthur R, Møller H, Garmo H, Holmberg L, Stattin P, Malmstrom H, et al Association between baseline serum glucose, triglycerides and total cholesterol, and prostate cancer risk categories. Cancer Med 2016;5: 1307–1318. 10.1002/cam4.665 26923095 PMC4924389

[5] Hanahan D, Weinberg RA. Hallmarks of cancer: the next generation. Cell 2011;144: 646–674. 10.1016/j.cell.2011.02.013 21376230

[6] Pinter M, Jain RK. Targeting the renin-angiotensin system to improve cancer treatment: Implications for immunotherapy. Sci Transl Med. 2017;9 10.1126/scitranslmed.aan5616 28978752 PMC5928511

[7] Sun H, Li T, Zhuang R, Cai W, Zheng Y. Do renin–angiotensin system inhibitors influence the recurrence, metastasis, and survival in cancer patients?: Evidence from a meta-analysis including 55 studies. Medicine. 2017;96: e6394 10.1097/MD.0000000000006394 28353566 PMC5380250

[8] Scott-Emuakpor J, Allot E, Johnson SA, Howard LE, Macias E, Freedland SJ, et al. Angiotensin receptor signaling and prostate tumor growth in mice. J Exp Ther Oncol. 2017; 11: 107–115. 28976133 PMC6311706

[9] Domińska K, Ochędalski T, Kowalska K, Matysiak-Burzyńska ZE, Płuciennik E, Piastowska-Ciesielska AW. A common effect of angiotensin II and relaxin 2 on the PNT1A normal prostate epithelial cell line. J Physiol Biochem. 2016;72: 381–392. 10.1007/s13105-016-0489-1 27119161

[10] Domińska K, Piastowska AW, Rebas E, Lachowicz-Ochedalska A. The influence of peptides from the angiotensin family on tyrosine kinase activity and cell viability in a human hormone-dependent prostate cancer line. Endokrynol Pol. 2009;60: 363–369. 19885807

[11] Holmes S, Griffith EJ, Musto G, Minuk GY. Antihypertensive medications and survival in patients with cancer: A population-based retrospective cohort study. Cancer Epidemiol. 2013;37: 881–885. 10.1016/j.canep.2013.09.001 24075077

[12] Poch MA, Mehedint D, Green DJ, Payne-Ondracek R, Fontham ET, Bensen JT, et al The association between calcium channel blocker use and prostate cancer outcome. Prostate. 2013;73: 865–872. 10.1002/pros.22632 23280547 PMC5826547

[13] Cardwell CR, Coleman HG, Murray LJ, O’Sullivan JM, Powe DG. Beta-blocker usage and prostate cancer survival: a nested case-control study in the UK Clinical Practice Research Datalink cohort. Cancer Epidemiol. 2014;38: 279–285. 10.1016/j.canep.2014.03.011 24786858

[14] Cardwell CR, Mc Menamin ÚC, Hicks BM, Hughes C, Cantwell MM, Murray LJ. Drugs affecting the renin-angiotensin system and survival from cancer: a population based study of breast, colorectal and prostate cancer patient cohorts. BMC Med. 2014;12: 28 10.1186/1741-7015-12-28 24521426 PMC3926686

[15] Lu H, Liu X, Guo F, Tan S, Wang G, Liu H, et al Impact of beta-blockers on prostate cancer mortality: a meta-analysis of 16,825 patients. Onco Targets Ther. 2015;8: 985–990. 10.2147/OTT.S78836 25995645 PMC4425323

[16] Grytli HH, Fagerland MW, Fosså SD, Taskén KA, Håheim LL. Use of beta-blockers is associated with prostate cancer-specific survival in prostate cancer patients on androgen deprivation therapy. Prostate. 2013;73: 250–260. 10.1002/pros.22564 22821802

[17] Grytli HH, Fagerland MW, Fosså SD, Taskén KA. Association between use of β-blockers and prostate cancer-specific survival: a cohort study of 3561 prostate cancer patients with high-risk or metastatic disease. Eur Urol. 2014;65: 635–641. 10.1016/j.eururo.2013.01.007 23351721

[18] Santala EEE, Rannikko A, Murtola TJ. Antihypertensive drugs and prostate cancer survival after radical prostatectomy in Finland–a nationwide cohort study. Int J Cancer. 2019; 144: 440–447. 10.1002/ijc.31802 30110124

[19] Siltari A, Murtola TJ, Talala K, Taari K, Tammela TJL, Auvinen A. Antihypertensive drugs and prostate cancer risk in a Finnish population-based cohort. Scand J Urol. 2019;52: 1–7. 10.1080/21681805.2018.1559882 30698056

[20] Kilpeläinen TP, Tammela TL, Malila N, Hakama M, Santti H, Määttänen L, et al Prostate cancer mortality in the Finnish randomized screening trial. J Natl Cancer Inst. 2013;105: 719–725. 10.1093/jnci/djt038 23479454

[21] Leinonen MK, Miettinen J, Heikkinen S, Pitkäniemi J, Malila N, et al Quality measures of the population-based finnish cancer registry indicate sound data quality for solid malignant tumours. Eur J Cancer. 2017;77: 31–39. 10.1016/j.ejca.2017.02.017 28350996

[22] Sarre S, Määttänen L, Tammela TLJ, Auvinen A, Murtola TJ. Postscreening follow-up of the Finnish Prostate Cancer Screening Trial on putative prostate cancer risk factors: vitamin and mineral use, male pattern baldness, pubertal development and non-steroidal anti-inflammatory drug use. Scand J Urol. 2016;50: 267–273. 10.3109/21681805.2016.1145734 26927237

[23] WHO ATC/DDD index 2016. http://www.whocc.no/atc_ddd_index/

[24] Veitonmäki T, Murtola TJ, Talala K, Taari K, Tammela T, Auvinen A. Non-Steroidal Anti-Inflammatory Drugs and Cancer Death in the Finnish Prostate Cancer Screening Trial. PLoS One 2016;11: e0153413 10.1371/journal.pone.0153413 eCollection 2016. 27100876 PMC4839624

[25] Ronquist G, Frithz G, Wang YH, Lindeborg T. Captopril may reduce biochemical (prostate-specific antigen) failure following radical prostatectomy for clinically localized prostate cancer. Scand J Urol Nephrol. 2009;43: 32–36. 10.1080/00365590802468875 18932051

[26] Alashkham A, Paterson C, Windsor P, Struthers A, Rauchhaus P, Nabi G. The incidence and risk of biochemical recurrence following radical radiotherapy for prostate cancer in men on angiotensin-converting enzyme inhibitors (ACEIs) or angiotensin receptor blockers (ARBs). Clin Genitourin Cancer. 2016;14: 398–405. 10.1016/j.clgc.2016.03.009 27053500

[27] Atlas SA. The renin-angiotensin aldosterone system: pathophysiological role and pharmacologic inhibition. J Manag Care Pharm. 2007;13: 9–20. 10.18553/jmcp.2007.13.s8-b.9 17970613 PMC10437584

[28] Mao Y, Xu X, Wang X, Zheng X, Xie L. Is angiotensin-converting enzyme inhibitors/angiotensin receptor blockers therapy protective against prostate cancer? Oncotarget. 2016;7: 6765–6773. 10.18632/oncotarget.6837 26760503 PMC4872747

[29] Shen J, Huang YM, Wang M, Hong XZ, Song XN, Zou X, et al Renin-angiotensin system blockade for the risk of cancer and death. J Renin Angiotensin Aldosterone Syst. 2016;17: 1470320316656679. 10.1177/1470320316656679 27402638 PMC5843874

[30] Assayag J, Pollak MN, Azoulay L. Post-diagnostic use of beta-blockers and the risk of death in patients with prostate cancer. Eur J Cancer. 2014;50: 2838–2845. 10.1016/j.ejca.2014.08.006 25204805

[31] Weberpals J, Jansen L, Carr PR, Hoffmeister M, Brenner H. Beta blockers and cancer prognosis—The role of immortal time bias: A systematic review and meta-analysis. Cancer Treat Rev. 2016;47: 1–11. 10.1016/j.ctrv.2016.04.004 27179912

[32] Bosco C, Wong C, Garmo H, Crawley D, Holmberg L, Hammar N, et al Drugs for metabolic conditions and prostate cancer death in men on GnRH agonists. BJU Int. 2018;121: 260–267. 10.1111/bju.14023 28940667

[33] Mäkinen T, Karhunen P, Aro J, Lahtela J, Määttänen L, Auvinen A. Assessment of causes of death in a prostate cancer screening trial. Int J Cancer. 2008;122: 413–417. 10.1002/ijc.23126 17935123

[34] Raval AD, Thakker D, Negi H, Vyas A, Kaur H, Salkini MW. Association between statins and clinical outcomes among men with prostate cancer: a systematic review and meta-analysis. Prostate Cancer Prostatic Dis. 2016;19: 151–162. 10.1038/pcan.2015.58 26782711

